# Supplementary material for: 12 new susceptibility loci for prostate cancer identified by genome-wide association study in Japanese population
Source: Nat Commun. 2019 Sep 27;10:4422. doi: 10.1038/s41467-019-12267-6 (PMC6764957; doi:10.1038/s41467-019-12267-6)
Supplement: Supplementary file 1 — Supplementary Information [file 41467_2019_12267_MOESM1_ESM.pdf]

Supplementary Information

12 New Susceptibility Loci for Prostate Cancer Identified by Genome-wide Association Study in Japanese Population

Takata, R. *et al.*

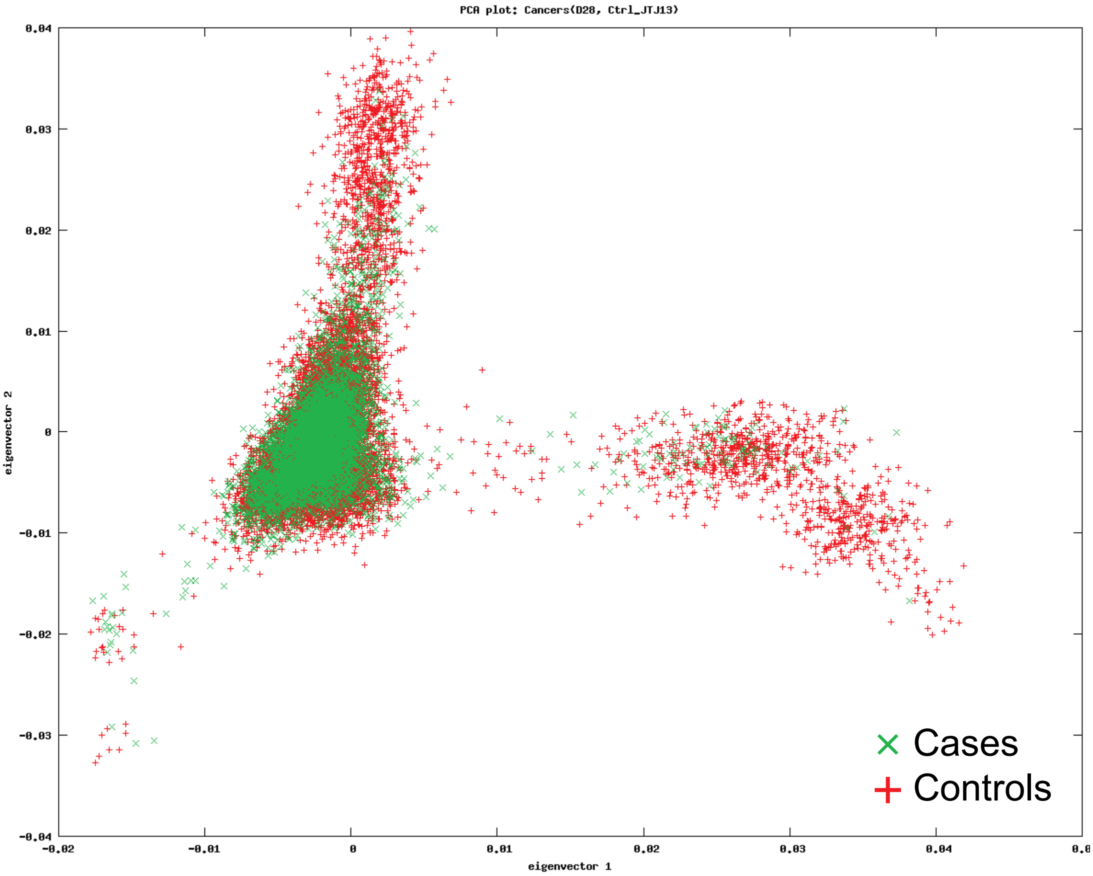

Supplementary Figure 1

Principal component analysis plot for the Japanese populations. The individuals were plotted in a two-dimensional graph, with the first (x axis) and the second (y axis) components of the Eigenvector factors.

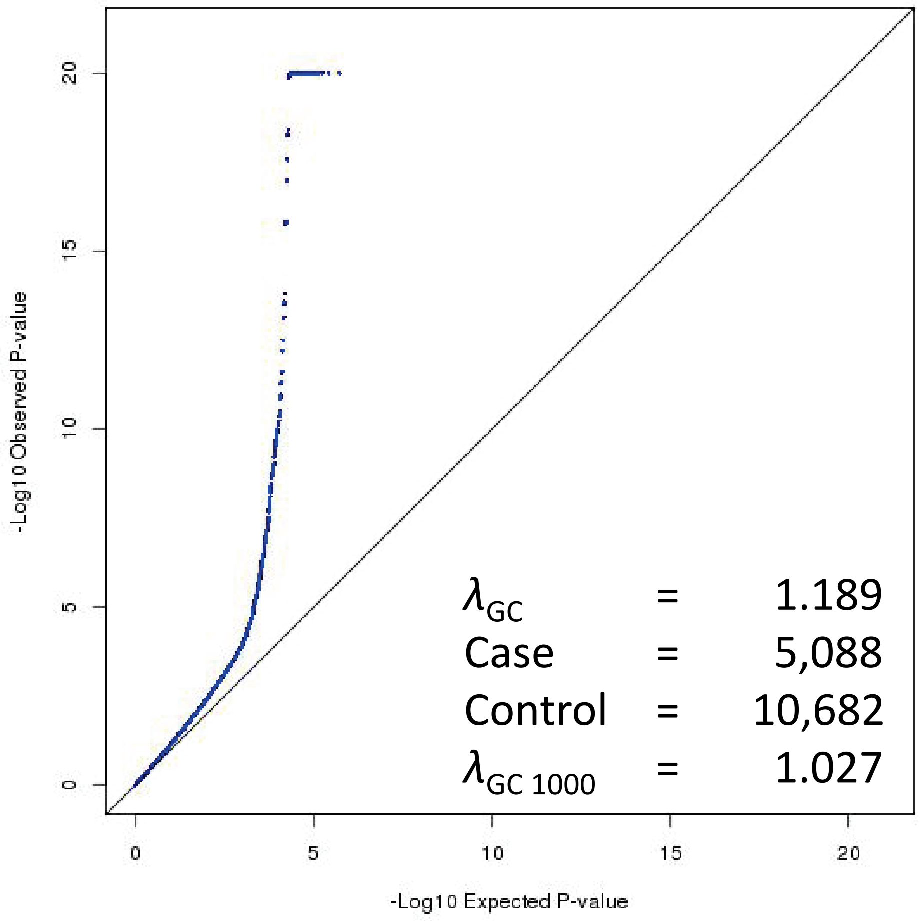

Supplementary Figure 2

Quantile-quantile plot of GWAS *P*-values for genotyped and imputed SNPs. Horizontal and vertical lines represent expected *P* values under null distribution and observed *P* values, respectively. The continuous line represents the expected distribution assuming no inflation of the statistics (y = x). $\lambda_{GC\ 1000}$ is the inflation of association statistics that would be expected in a study of 1,000 cases and 1,000 controls.

Supplementary Figure 3

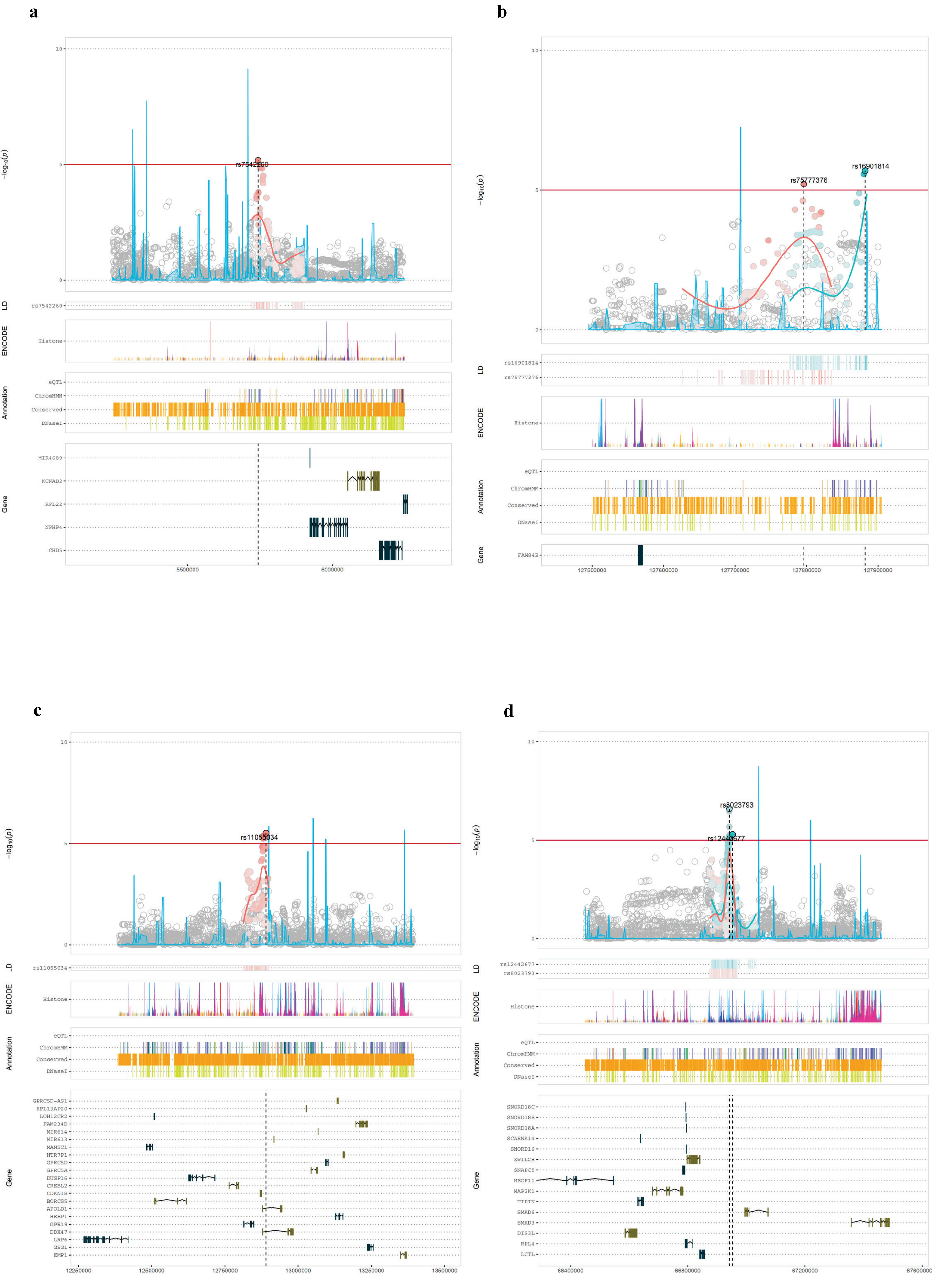

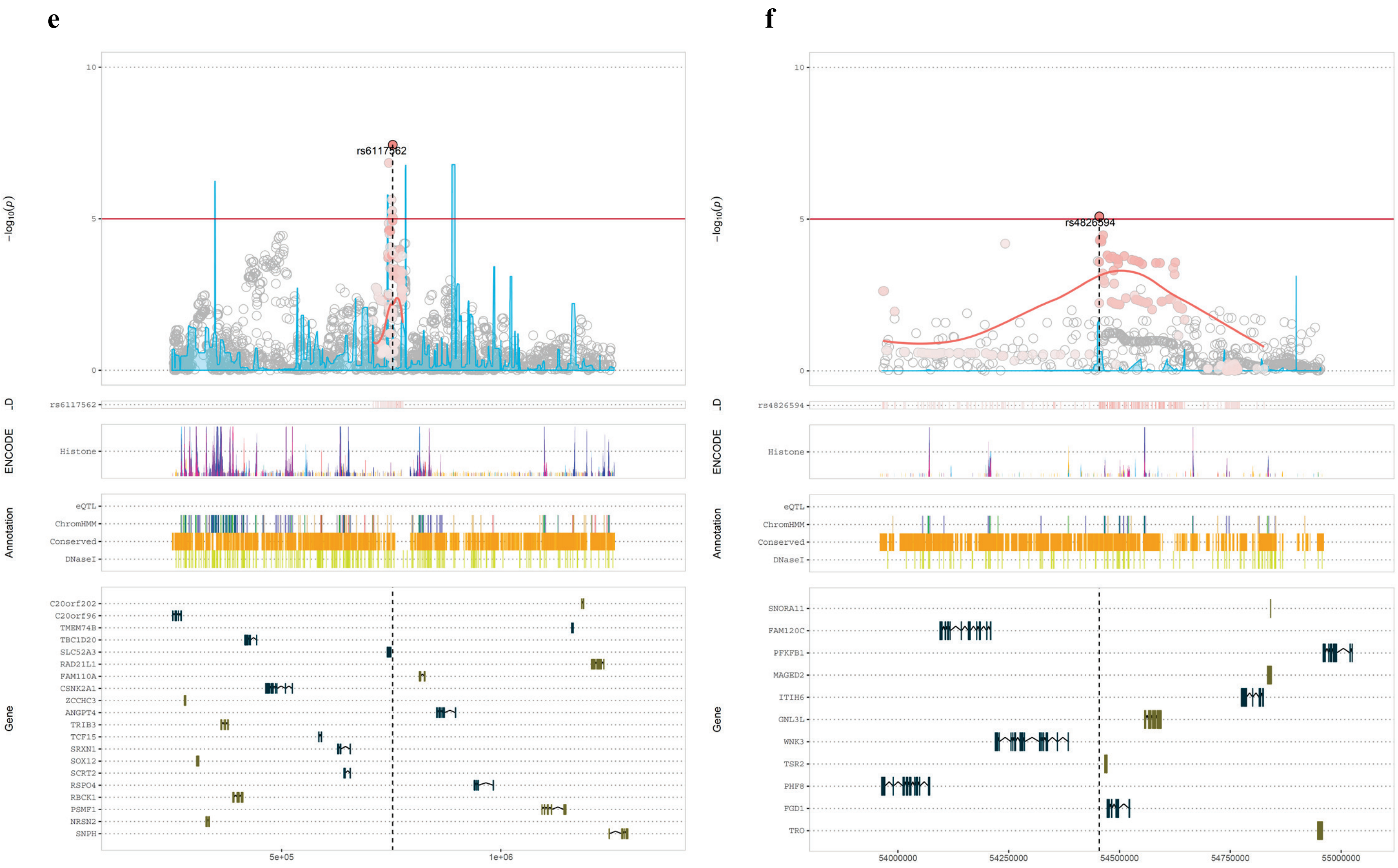

Supplementary Figure 3

Locus Explorer plots of novel seven GWAS loci. **a** rs7542260 at chr.1. **b** rs75777376 and rs16901814 at chr.8. **c** rs11055034 at chr.12. **d** rs8023793 at chr.15. **e** rs6117562 at chr.20. **f** rs4826594 at chr.X. The regional association plot (-log10(*P*) panel) depicts variant *P*-values relative to chromosomal position. Variants in linkage disequilibrium with the novel lead SNP(s) at r^2 ≥0.1 according to the 1000 Genomes JPT population are shaded in the Manhattan plot and linkage disequilibrium track (LD panel), with darker colour denoting stronger correlation with the lead variant. Lower sections of the plot indicate the relative positions of genes and selected biological annotations. Annotations displayed are: histone modifications in ENCODE tier 1 cell lines (Histone track), the positions of variants that are eQTLs with prostate tumor expression in TCGA prostate adenocarcinoma samples (eQTL track), chromatin state categorisations in the PrEC cell-line by ChromHMM (ChromHMM track), the position of conserved element peaks (Conserved track) and the position of DNaseI hypersensitivity site peaks in ENCODE prostate cell-lines (DNaseI track). Genes on the positive and negative strand are denoted by brown and turquoise color respectively (Gene track). The horizontal axis represents genomic coordinates in the hg19 reference genome.

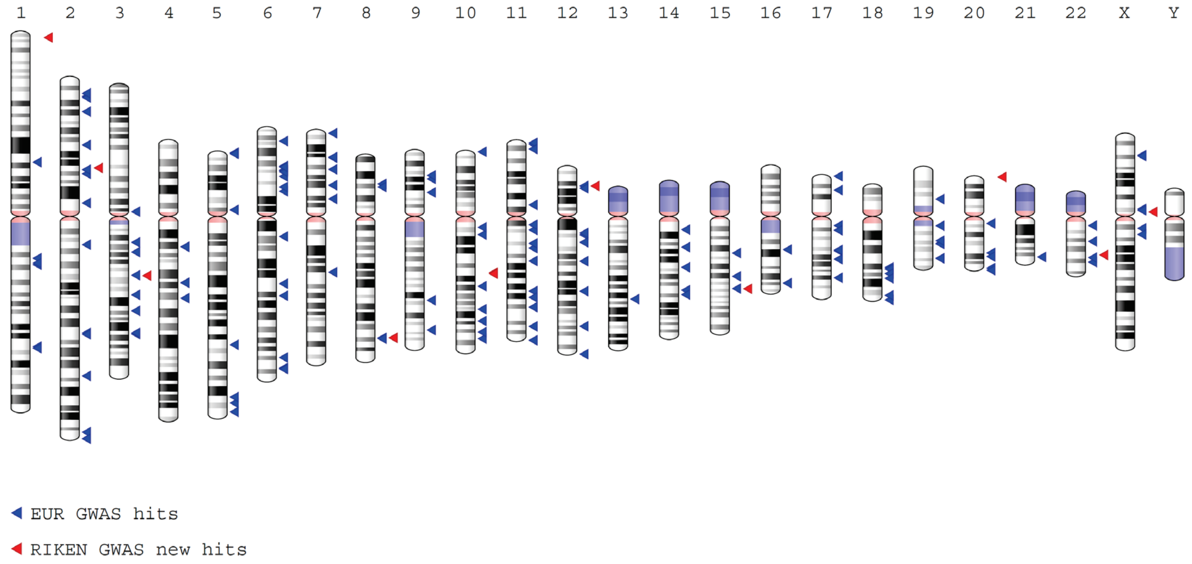

Supplementary Figure 4

PCa susceptibility loci identified in European GWAS and the novel loci identified in the present study. The PCa susceptibility loci reported from European GWAS (blue triangle) and the novel loci identified in the present study (red triangle) are denoted on a chromosome map.

Supplementary Figure 5

a

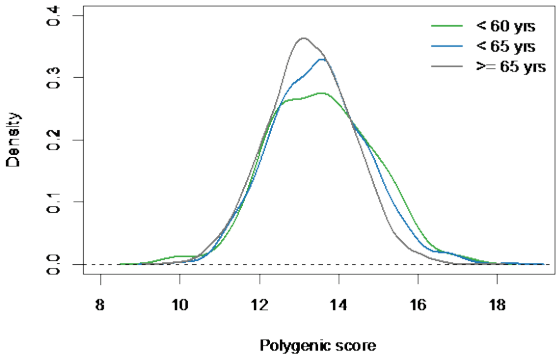

< 60 yrs vs others: $P = 0.02395$

< 65 yrs vs others: $P = 3.24 \times 10^{-7}$

b

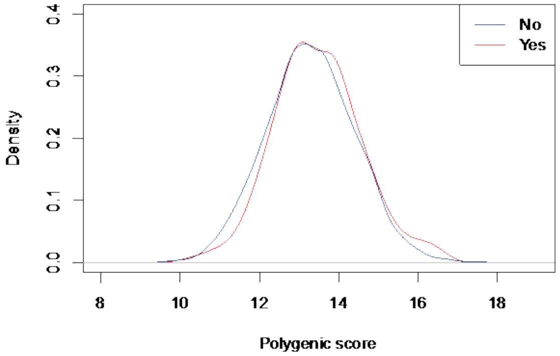

$P = 0.02395$

c

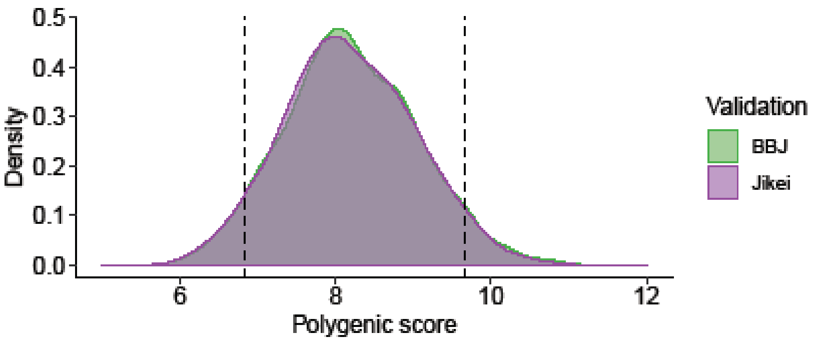

d

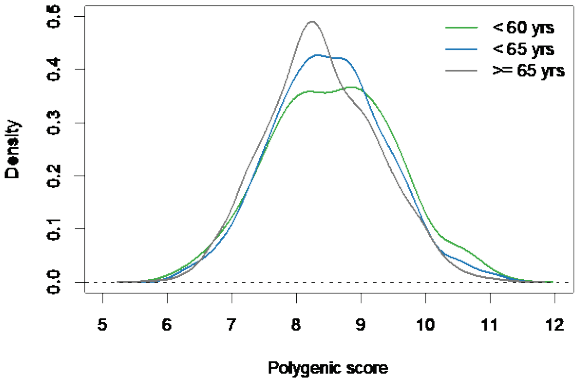

e

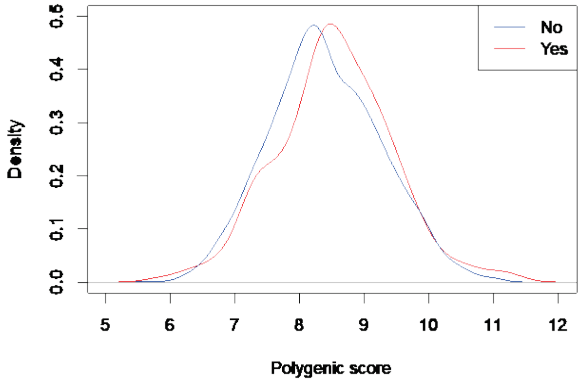

Supplementary Figure 5

The PRS distribution for prostate cancer in Japanese population. **a** The distribution of the PRS incorporating 150 SNPs by the age at diagnosis of PCa in the GWAS cases (*n*=4,762). **b** The distribution of PRS incorporating 150 SNPs by the presence of PCa family history in the GWAS cases (*n*=4,762). Density was estimated using the Gaussian kernel. Green, younger than 60 years (*n*=129); blue, younger than 65 years (*n*=781); gray, 65 years or older (*n*=3,852). **c** The PRS distribution of the PCa cases in the replication cohorts BBJ (*n*=2,368) and JIKEI (*n*=2,218). Density was estimated using the Gaussian kernel. The 5% higher and lower percentiles are shown as dotted lines. **d** The PRS distribution by the age at diagnosis of PCa in the BBJ cases (*n*=2,523). Density was estimated using the Gaussian kernel. Green, younger than 60 years (*n*=155); blue, younger than 65 years (*n*=530); gray, 65 years or older (*n*=1,838). **e** The PRS distribution by the presence of PCa family history in the BBJ cases (*n*=2,386). Density was estimated using the Gaussian kernel. Red, positive PCa family history (*n*=232); blue, negative PCa family history (*n*=2,154).

Supplementary Table 1 Clinical Characteristics of the Subjects.

		GWAS case (%)	BBJ replication case (%)	JIKEI case(%)	GWAS control(%)	3BJ replication control(%
Age at diagnosis	<50	3 (0.1)	4 (0.2)	18 (0.7)	2701 (25.3)	12978 (17.7)
	50–54	33 (0.6)	28 (1.2)	76 (2.9)	1434 (13.4)	5454 (7.4)
	55–59	93 (1.8)	123 (5.2)	216 (8.4)	1904 (17.8)	9049 (12.4)
	60–64	652 (12.8)	375 (15.7)	492 (19.1)	1923 (18.0)	10354 (14.1)
	65–69	1075 (21.1)	542 (22.7)	596 (23.1)	2025 (19.0)	11235 (15.3)
	70–74	1342 (26.4)	649 (27.2)	558 (21.6)	646 (6.0)	11069 (15.1)
	75–79	917 (18.0)	429 (18.0)	233 (9.0)	48 (0.4)	7876 (10.8)
	80–84	385 (7.6)	172 (7.2)	25 (1.0)	1 (0.0)	3676 (5.0)
	85–89	115 (2.3)	42 (1.8)	3 (0.1)	0 (0.0)	1223 (1.7)
	90+	18 (0.4)	4 (0.2)	1 (0.0)	0 (0.0)	303 (0.4)
	N.A.	455 (8.9)	18 (0.8)	364 (14.1)	0 (0.0)	44 (0.0)
	Mean ± S.D.	71.2 ± 6.8	70.2 ± 7.1	66.7 ± 6.7	56.6± 9.9	61.8 ± 14.3
Family history of PCa	Yes	272 (5.3)	232 (9.7)	0 (0.0)		
	No	4621 (90.8)	2154 (90.3)	0 (0.0)		
	N.A.	195 (3.8)	0 (0.0)	2582 (100.0)		
PSA (ng/mL)	≤4	131 (2.6)	29 (1.2)	67 (2.6)		
	4–10	869 (17.1)	1061 (44.5)	1371 (53.1)		
	10–20	510 (10.0)	563 (23.6)	530 (20.5)		
	>20	709 (13.9)	678 (28.4)	243 (9.4)		
	N.A.	2869 (56.4)	55 (2.3)	371 (14.4)		
T stage	T0	9 (0.2)	0 (0.0)	3 (0.1)		
	T1	516 (10.1)	519 (21.8)	930 (36.0)		
	T2	792 (15.6)	941 (39.4)	758 (29.4)		
	T3	406 (8.0)	324 (13.6)	414 (16.0)		
	T4	88 (1.7)	74 (3.1)	10 (0.4)		
	N.A.	3277 (64.4)	528 (22.1)	467 (18.1)		
N stage	N0	1636 (32.2)	1717 (72.0)	0 (0.0)		
	N1	94 (1.8)	122 (5.1)	0 (0.0)		
	N.A.	3358 (66.0)	547 (22.9)	2582 (100.0)		
M stage	M0	1582 (31.1)	1663 (69.7)	0 (0.0)		
	M1	125 (2.5)	146 (6.1)	0 (0.0)		
	N.A.	3381 (66.5)	577 (24.2)	2582 (100.0)		
Gleason Score	≤6	1293 (25.4)	672 (28.2)	609 (23.6)		
	7	1109 (21.8)	800 (33.5)	1188 (46.0)		
	≥8	880 (17.3)	736 (30.8)	412 (16.0)		
	N.A.	1806 (35.5)	178 (7.5)	373 (14.4)		

Supplementary Table 2 101 SNPs analyzed for the replication study

SNP ID	Chr	Position	Effect Allele	NonEffect Allele	Case Frequency	Control Frequency	RSQR[a]	OR[b]	*P* -value	Nearby genes
rs7542260	1	5743196	C	T	0.892	0.910	0.980	0.832	6.64.E-06	*MIR4417*
rs75577813	1	31176551	T	A	0.844	0.827	0.770	1.194	3.13.E-06	*MATN1*
rs1149035	1	31178783	C	T	0.712	0.687	0.996	1.138	1.38.E-06	*MATN1*
rs1149036	1	31179677	C	T	0.539	0.512	0.993	1.126	1.34.E-06	*MATN1*
rs17127969	1	66220441	G	C	0.895	0.879	0.599	1.259	5.91.E-06	*PDE4B*
rs77631267	1	96451709	T	C	0.972	0.979	0.611	0.640	4.73.E-06	*UBE2WP1*
rs680823	1	110848727	C	G	0.708	0.681	0.998	1.127	6.31.E-06	*LOC440600*
rs10200833	2	43673316	G	C	0.712	0.675	0.950	1.177	2.12.E-09	*THADA*
rs6544670	2	43771237	T	A	0.897	0.886	0.366	1.357	4.14.E-06	*THADA*
rs11676567	2	62733253	T	C	0.612	0.643	0.953	0.869	5.17.E-08	*TMEM17*
rs12622031	2	62741388	C	A	0.661	0.697	0.977	0.865	3.65.E-08	*RPL37P13*
rs11125927	2	62752975	A	G	0.738	0.774	0.980	0.837	4.74.E-10	*RPL21P37*
rs58235267	2	63277843	G	C	0.738	0.716	0.901	1.143	4.11.E-06	*OTX1*
rs11890276	2	63366191	T	C	0.744	0.719	0.994	1.149	5.91.E-07	*WDPCP*
rs114780236	2	63789049	G	A	0.848	0.826	0.989	1.166	5.49.E-06	*WDPCP*
rs72806089	2	63906145	A	T	0.836	0.815	0.915	1.166	6.84.E-06	*RPL27P6*
rs79600468	2	150358312	T	C	0.814	0.831	0.641	0.833	3.02.E-06	*LYPD6*
rs10469655	2	150411229	A	T	0.878	0.896	0.948	0.829	1.51.E-06	*MMADHC*
rs838437	2	232580258	C	T	0.625	0.654	0.977	0.890	5.43.E-06	*PTMA*
rs55702004	3	25398113	A	G	0.899	0.884	0.689	1.267	9.63.E-07	*LOC100505947*
rs80041270	3	86634986	A	G	0.869	0.841	0.725	1.253	6.98.E-08	*VGLL3*
rs73205316	3	127592030	G	A	0.837	0.852	0.610	0.827	5.94.E-06	*KBTBD12*
rs2878602	3	127709698	C	T	0.634	0.663	0.955	0.881	1.13.E-06	*KBTBD12*
rs146054659	3	127777865	A	C	0.981	0.985	0.332	0.493	8.62.E-06	*SEC61A1*
rs62270939	3	128189071	C	G	0.972	0.983	0.699	0.527	1.29.E-11	*DNAJB8-AS1*
rs2659689	3	128203006	T	G	0.490	0.516	0.973	0.874	5.78.E-08	*GATA2*
rs73862213	3	128217499	A	G	0.908	0.929	0.996	0.754	1.71.E-10	*LOC100506995*
chr3_128292393	3	128292393	G	A	0.971	0.977	0.378	0.531	9.26.E-08	*C3orf27*
rs9877767	3	128492372	C	T	0.979	0.985	0.397	0.469	1.92.E-08	*RAB7A*
rs62269375	3	145469759	T	C	0.918	0.903	0.989	1.214	9.28.E-06	*GM2AP1*
rs9684980	4	58386872	T	C	0.926	0.915	0.424	1.428	4.95.E-07	*RPS26P24*
rs79913506	4	58463352	A	G	0.982	0.978	0.324	2.086	6.59.E-06	*RPS26P24*
rs184519554	4	58487183	G	C	0.887	0.874	0.579	1.262	3.73.E-06	*RPS26P24*
rs28479946	4	95596744	A	T	0.744	0.771	0.863	0.872	7.58.E-06	*PDLIM5*
rs140589300	4	134260340	G	A	0.952	0.938	0.680	1.352	6.99.E-06	*PCDH10*
rs138819533	4	134599768	G	A	0.962	0.952	0.437	1.578	1.96.E-06	*PCDH10*
rs72804215	5	123404120	T	G	0.847	0.869	0.821	0.839	3.69.E-06	*KRT18P16*
rs117276599	6	21313499	C	T	0.930	0.941	0.575	0.751	8.10.E-06	*CDKAL1*
rs241819	6	100910798	G	A	0.594	0.618	1.000	0.894	6.83.E-06	*SIM1*
rs79736035	6	117372016	C	T	0.851	0.863	0.359	0.754	1.29.E-06	*RPS29P13*
rs553058	6	117374977	T	A	0.451	0.485	0.952	0.875	1.04.E-07	*RPS29P13*
rs188059637	6	136003357	A	T	0.963	0.951	0.986	1.320	9.10.E-06	*LINC00271*
rs10232385	7	21185228	T	C	0.703	0.676	0.999	1.128	5.27.E-06	*ASS1P11*
rs76282104	7	25894349	G	T	0.989	0.983	0.851	1.722	8.44.E-06	*LOC100506236*
rs13246464	7	27684029	C	G	0.903	0.917	0.754	0.800	4.77.E-06	*HIBADH*
rs150873210	7	124180638	A	C	0.943	0.951	0.982	0.784	8.02.E-06	*RPS2P31*
rs62481516	7	124207578	T	C	0.943	0.951	0.991	0.786	8.20.E-06	*RPS2P31*
rs62478329	7	124314512	C	T	0.942	0.950	0.941	0.778	4.58.E-06	*GPR37*
rs73443807	7	124347349	T	C	0.941	0.949	0.981	0.786	5.72.E-06	*GPR37*
rs35633617	7	150675562	A	G	0.880	0.861	0.998	1.181	6.38.E-06	*KCNH2*
rs141301022	8	23276085	G	A	0.951	0.960	0.730	0.686	4.70.E-08	*ENTPD4*
rs181545207	8	24329446	T	C	0.958	0.949	0.752	1.361	9.13.E-06	*ADAM7*
rs75777376	8	127796183	T	C	0.896	0.908	0.528	0.779	6.08.E-06	*FAM84B*
rs16901814	8	127881952	G	A	0.854	0.833	0.993	1.176	2.00.E-06	*SRRM1P1*
rs12341053	9	31095621	T	C	0.799	0.780	0.989	1.146	6.51.E-06	*LOC100533658*
rs190296145	9	101773862	A	G	0.628	0.607	0.980	1.119	8.43.E-06	*COL15A1*
rs4837088	9	129223653	T	A	0.893	0.906	0.800	0.817	5.98.E-06	*FAM125B*
rs150059818	10	80254699	G	A	0.850	0.831	0.818	1.204	5.53.E-07	*LOC100132987*
rs56231799	10	80258348	T	C	0.792	0.766	0.965	1.180	4.49.E-08	*LOC100132987*
rs77911174	10	80826833	A	G	0.604	0.643	0.957	0.853	6.14.E-10	*LOC283050*
rs11196775	10	116279023	T	G	0.800	0.826	0.990	0.870	7.61.E-06	*ABLIM1*
rs11199697	10	122758389	A	C	0.831	0.854	1.000	0.859	4.83.E-06	*WDR11*
rs142721250	11	21513536	A	G	0.973	0.980	0.652	0.632	1.39.E-06	*NELL1*
rs116924860	11	27614016	G	A	0.982	0.987	0.538	0.542	5.04.E-06	*BDNF-AS1*
rs3812767	11	36293835	C	G	0.639	0.663	0.997	0.888	3.56.E-06	*COMMD9*
rs144783953	11	36308881	A	G	0.556	0.532	0.868	1.137	9.97.E-07	*COMMD9*
rs1900337	11	36357037	A	G	0.861	0.841	1.000	1.170	6.44.E-06	*PRR5L*
rs149911096	11	58816706	G	C	0.853	0.870	0.624	0.809	2.08.E-06	*LOC283194*
rs10735041	12	5013308	A	G	0.701	0.663	0.973	1.153	1.02.E-07	*KCNA1*
rs12228019	12	5013456	C	A	0.764	0.735	0.961	1.159	3.37.E-07	*KCNA1*
rs993018	12	5037942	A	G	0.904	0.890	0.823	1.222	7.66.E-06	*KCNA1*
rs2855712	12	12031894	C	G	0.580	0.603	0.836	0.877	1.38.E-06	*ETV6*
rs11055034	12	12890626	C	A	0.601	0.577	0.999	1.123	3.11.E-06	*APOLD1*

rs187245946	12	53229473	C	T	0.976	0.983	0.448	0.540	2.95.E-07	*KRT79*
rs142025399	12	53315665	C	T	0.962	0.973	0.603	0.585	2.80.E-10	*KRT8*
rs795401	12	61389065	A	C	0.737	0.711	0.852	1.154	1.80.E-06	*PGBD3P1*
rs145628451	12	61398726	A	C	0.582	0.557	0.850	1.128	7.27.E-06	*PGBD3P1*
rs4842687	12	90156377	A	G	0.740	0.721	0.835	1.144	7.98.E-06	*MRPL2P1*
rs57298504	12	97889116	C	T	0.887	0.868	0.985	1.183	9.86.E-06	*RMST*
rs74648385	13	51225503	C	T	0.961	0.963	0.498	0.663	8.15.E-06	*DLEU7*
rs35366543	14	62869613	G	A	0.823	0.840	0.939	0.860	6.39.E-06	*LOC100129782*
rs7169790	15	43671639	T	A	0.639	0.622	0.843	1.134	4.66.E-06	*TUBGCP4*
rs8023793	15	66942093	A	C	0.595	0.559	0.997	1.137	2.56.E-07	*SMAD6*
rs12442677	15	66952824	C	T	0.805	0.775	0.864	1.160	5.22.E-06	*SMAD6*
rs237342	17	12428982	G	A	0.592	0.616	0.640	0.872	9.13.E-06	*FLJ34690*
rs12150178	17	36543271	G	A	0.843	0.826	0.349	1.301	3.40.E-06	*SOCS7*
rs8067707	17	36570808	A	G	0.798	0.770	0.888	1.161	2.61.E-06	*SOCS7*
rs7247729	19	19837946	A	G	0.702	0.680	0.813	1.142	6.31.E-06	*ZNF14*
rs3746804	20	744415	G	A	0.697	0.669	1.000	1.149	1.44.E-07	*C20orf54*
rs58279463	20	750499	C	A	0.790	0.770	0.973	1.153	2.35.E-06	*C20orf54*
rs6117562	20	753310	G	A	0.485	0.516	0.989	0.874	3.64.E-08	*C20orf54*
rs55682426	20	9800385	A	G	0.670	0.696	0.998	0.877	5.60.E-07	*PAK7*
rs761227	20	9806004	T	C	0.587	0.613	0.950	0.886	2.29.E-06	*PAK7*
rs2253231	21	16352281	C	T	0.591	0.555	1.000	1.152	1.24.E-08	*NRIP1*
rs1997577	21	16371102	A	T	0.651	0.619	0.978	1.156	1.89.E-08	*NRIP1*
rs7282769	21	16381531	A	G	0.706	0.679	0.984	1.143	6.03.E-07	*NRIP1*
rs34567417	22	37257177	G	A	0.935	0.948	0.886	0.772	1.82.E-06	*NCF4*
rs138426	22	38871891	A	G	0.717	0.694	0.746	1.148	9.32.E-06	*KDELR3*
rs117693861	22	38993476	A	G	0.966	0.958	0.378	1.687	1.20.E-06	*LOC646851*
rs138708	22	39138332	G	A	0.855	0.831	0.983	1.172	3.74.E-06	*SUN2*
rs4826594	X	54454406	G	A	0.482	0.525	0.967	0.924	8.21.E-06	*TSR2*

[a] RSQR, imputation accuracy. SNPs were imputed in the GWAS.

[b] Non effect alleles were considered as reference.

Supplementary Table 3 eQTL analysis indicated four SNPs were associated with expression of nearby genes

SNP	Gene name	variant_id	tss_distance[a]	pval_nominal[b]	slope[c]	slope_se[d]
rs16901814	*FAM84B*	8_127881952_G_A_b37	311314	2.04.E-02	0.433	0.182
rs4554825	*ZMIZ1-AS1*	10_80244623_C_T_b37	-583029	3.61.E-02	0.408	0.191
rs6117562	*CSNK2A1*	20_753310_G_A_b37	228845	2.76.E-02	0.269	0.119
rs6117562	*FAM110A*	20_753310_G_A_b37	-61048	1.04.E-02	0.176	0.067

[a]Distance between variant and transcription start site.

[b]Nominal p-value associated with the variant for this gene.

[c]Regression slope.

[d]Standard error of the regression slope.

Supplementary Table 4   Allele frequency of the 12 novel SNPs in European and Japanese population

SNP ID	1000 Genomes				Hapmap			
	European		Japanese		European		Japanese	
rs7542260	C: 0.955 (189)	T: 0.045 (9)	C: 0.856 (178)	T: 0.144 (30)	C: 0.947 (108)	T: 0.053 (6)	C: 0.845 (71)	T: 0.155 (13)
rs11125927	G: 0.111 (22)	A: 0.889 (176)	G: 0.245 (51)	A: 0.755 (157)	G: 0.100 (12)	A: 0.900 (108)	G: 0.233 (21)	A: 0.767 (69)
rs73862213	A: 0.924 (183)	G: 0.076 (15)	A: 0.904 (188)	G: 0.096 (20)				
rs75777376	T: 0.995 (197)	C: 0.005	T: 0.894 (186)	C: 0.106 (22)				
rs16901814	G: 0.854 (169)	A: 0.146 (29)	G: 0.803 (167)	A: 0.197 (41)	G: 0.858 (194)	A: 0.142 (32)	G: 0.814 (140)	A: 0.186 (32)
rs4554825	C: 0.944 (187)	T: 0.056 (11)	C: 0.774 (161)	T: 0.226 (47)	C: 0.924 (109)	T: 0.076 (9)	C: 0.767 (69)	T: 0.233 (21)
rs77911174	A: 0.904 (179)	G: 0.096 (19)	A: 0.644 (134)	G: 0.356 (74)				
rs11055034	C: 0.712 (141)	A: 0.288 (57)	C: 0.529 (110)	A: 0.471 (98)	C: 0.704 (159)	A: 0.296 (67)	C: 0.530 (89)	A: 0.470 (79)
rs8023793	C: 0.470 (93)	A: 0.530 (105)	C: 0.404 (84)	A: 0.596 (124)				
rs6117562	G: 0.773 (153)	A: 0.227 (45)	G: 0.587 (122)	A: 0.413 (86)	G: 0.788 (178)	A: 0.212 (48)	G: 0.599 (103)	A: 0.401 (69)
rs138708	G: 0.985 (195)	A: 0.015 (3)	G: 0.803 (167)	A: 0.197 (41)	G: 1.000 (116)	A: 0.000 (0)	G: 0.802 (69)	A: 0.198 (17)
rs4826594	G: 0.919 (137)	A: 0.081 (12)	G: 0.539 (82)	A: 0.461 (70)	G: 0.916 (207)	A: 0.084 (19)	G: 0.517 (89)	A: 0.483 (83)

Supplementary Table 5 Independency of association of the new loci by conditional analysis

Marker	Referrence	*P* -value	
		GWAS	Conditional analysis
rs11125927	rs721048	4.74×10^{-10}	1.02×10^{-09}
rs73862213	rs10934853	1.71×10^{-10}	1.88×10^{-10}
rs75777376	rs12543663	6.08×10^{-06}	5.36×10^{-06}
rs16901814	rs12543663	2.00×10^{-06}	1.91×10^{-06}
rs4554825	rs77911174	1.06×10^{-07}	2.34×10^{-08}
rs4826594	rs5945619	8.21×10^{-06}	1.10×10^{-05}

Supplementary Table 6 Japanese PCa susceptibility SNPs that might be in the same lesions of reported loci.

	SNP ID	Chr	Position	Effect Allele	NonEffect Allele		Case Frequency	Control Frequency	RSQR[a]	OR[b]	95% CI	*P* -value	Nearby genes
3	rs114780236	2	63789049	G	A	GWAS	0.848	0.826	0.989	1.166	(1.098-1.234)	$5.49{\times}10^{-06}$	*WDPCP*
						Replication	0.853	0.829	-	1.204	(1.144-1.264)	$4.46{\times}10^{-10}$	
						Meta	-	-	-	1.187	-	$\mathbf{1.74{\times}10^{-14}}$	
10	rs4842687	12	90156377	A	G	GWAS	0.740	0.721	0.835	1.144	(1.084-1.204)	$7.98{\times}10^{-06}$	-
						Replication	0.780	0.756	-	1.142	(1.092-1.192)	$1.70{\times}10^{-07}$	
						Meta	-	-	-	1.143	-	$\mathbf{5.61{\times}10^{-12}}$	

[a]RSQR, imputation accuracy. SNPs were imputed in the GWAS.

[b]Non effect alleles were considered as reference.

Supplementary Table 7 The validation rate for the SNPs discovered using Asian cohort

Original	Japanese GWAS		Concordance
Race	*P* >0.05	*P* <0.05	Rate
European	41	34	0.46
Asian	2	8	0.80

Supplementary Table 8   82 SNPs used for polygenic risk score in Japanese GWAS and replication study

SNP ID	chr	position	Risk allele	Non-risk allele	OR[a] or DOSE[a]	genotyped/imputed	PRS for replication study	Related or nearby gene
rs7542260	1	5,743,196	T	C	1.186	imputed	〇	*MIR4417*
rs1110165	1	153,913,699	G	T	1.070	genotyped	X	*DENND4B*
rs4245739	1	204,518,842	A	C	1.154	genotyped	〇	*MDM4*
rs62106670	2	8,597,123	T	C	1.103	imputed	X	*LINC00299*
rs13385191	2	20,888,265	G	A	1.106	genotyped	〇	*C2orf43*
rs1465618	2	43,553,949	T	C	1.137	genotyped	〇	*THADA*
rs11125927	2	62,752,975	G	A	1.163	imputed	〇	*RPL21P37*
rs114780236	2	63,789,049	G	A	1.187	imputed	〇	*WDPCP*
rs10187424	2	85,794,297	T	C	1.083	genotyped	〇	*GGCX*
rs11691517	2	111,893,096	T	G	1.085	imputed	X	*BCL2L11*
rs12621278	2	173,311,553	A	G	1.182	genotyped	〇	*ITGA6*
rs34925593	2	174,234,547	C	T	1.081	imputed	X	*CDCA7*
rs2349070	2	202,130,308	C	A	1.056	imputed	X	*CASP8*
rs2292884	2	238,443,226	G	A	1.062	genotyped	〇	*MLPH*
rs2660753	3	87,110,674	T	C	1.178	genotyped	〇	*VGLL3*
rs2055109	3	87,467,332	C	T	1.195	imputed	〇	*FOU1F1*
rs7611694	3	113,275,624	A	C	1.073	imputed	〇	*SIDT1*
rs73862213	3	128,217,499	G	A	1.314	imputed	〇	*LOC100506995*
rs6763931	3	141,102,833	A	G	1.052	genotyped	〇	*ZBTB38*
rs12500426	4	95,514,609	A	C	1.051	genotyped	〇	*PDLIM5*
rs2242652	5	1,280,028	G	A	1.146	imputed	〇	*TERT*
rs12653946	5	1,895,829	T	C	1.225	genotyped	〇	*IRX4*
rs1983891	6	41,536,427	T	C	1.145	genotyped	〇	*FOXP4*
rs4711748	6	43,694,598	T	C	1.063	imputed	X	*LOC100132242*
rs9443189	6	76,495,882	A	G	1.097	imputed	〇	*MYO6*
rs339331	6	117,210,052	T	C	1.222	genotyped	〇	*RFX6*
rs1933488	6	153,441,079	A	G	1.068	genotyped	〇	*RGS17*
rs9364554	6	160,833,664	T	C	1.053	genotyped	〇	*SLC22A3*
rs12155172	7	20,994,491	A	G	1.084	genotyped	〇	*LINC01162/SP8*
rs10486567	7	27,976,563	G	A	1.195	genotyped	〇	*JAZF1*
rs17621345	7	40,875,192	A	C	1.245	genotyped	X	*C7orf10*
rs2928679	8	23,438,975	A	G	1.124	genotyped	〇	*NKX3.1*
rs1512268	8	23,526,463	T	C	1.129	genotyped	〇	*NKX3.1*
rs75777376	8	127,796,183	C	T	1.325	imputed	〇	*FAM84B*
rs16901814	8	127,881,952	G	A	1.167	genotyped	〇	*SRRM1P1*

rs10086908	8	128,011,937	T	C	1.269	genotyped	○	*8q24(Block1)*
rs16901979	8	128,124,916	A	C	1.429	imputed	○	*PRNCR1/8q24(Region2)*
rs620861	8	128,335,673	G	A	1.061	imputed	○	*8q24(Block3/Region3)*
rs6983267	8	128,413,305	G	T	1.156	genotyped	○	*8q24(Block4/Region3)*
rs1447295	8	128,485,038	A	C	1.506	genotyped	○	*CASC6*
rs7837688	8	128,539,360	T	G	1.727	genotyped	○	*8q24(Block5/Region1)*
rs1182	9	132,576,060	A	C	1.063	imputed	X	*TOR1A*
rs10993994	10	51,549,496	T	C	1.164	genotyped	○	*MSMB*
rs4554825	10	80,244,623	C	T	1.142	genotyped	○	*LOC100130698*
rs77911174	10	80,826,833	G	A	1.164	imputed	○	*LOC283050*
rs1935581	10	90,195,149	C	T	1.060	imputed	X	*RNLS*
rs7094871	10	114,712,154	G	C	1.078	imputed	X	*TCF7L2*
rs2252004	10	122,844,709	C	A	1.151	imputed	○	*FGFR2/WDR11*
rs61890184	11	7,547,587	A	G	1.148	imputed	X	*PPFIBP2*
rs1938781	11	58,915,110	G	A	1.116	imputed	○	*FAM111A*
rs7931342	11	68,994,497	G	T	1.065	genotyped	○	*MYEOV/TPCN2*
rs17245270	11	76,157,489	A	C	1.115	imputed	X	*C11orf30*
rs11055034	12	12,890,626	C	A	1.118	genotyped	○	*APOLD1*
rs902774	12	53,273,904	T	C	1.142	genotyped	○	*KRT78/KRT8*
rs7968403	12	65,012,824	T	C	1.103	imputed	X	*RASSF3*
rs4842687	12	90,156,377	A	G	1.143	imputed	○	*MRPL2P1*
rs1270884	12	114,685,571	A	G	1.093	imputed	○	*TBX5/RBM19*
rs7295014	12	133,067,989	G	A	1.125	imputed	X	*FBRSL1*
rs9600079	13	73,728,139	T	G	1.145	genotyped	○	*KLF5*
rs11629412	14	37,138,294	C	G	1.088	imputed	X	*PAX9*
rs7153648	14	61,122,526	C	G	1.072	imputed	○	*SIX1*
rs58262369	14	64,693,912	T	C	1.203	imputed	○	*ESR2*
rs8014671	14	71,092,256	G	A	1.084	genotyped	○	*TTC9*
rs4924487	15	40,922,915	C	G	1.057	imputed	X	*CASC5*
rs8023793	15	66,942,093	A	C	1.198	imputed	○	*SMAD6*
rs684232	17	618,965	C	T	1.077	genotyped	○	*VPS53*
rs11649743	17	36,074,979	G	A	1.147	genotyped	○	*HNF1B*
rs4430796	17	36,098,040	A	G	1.365	genotyped	○	*HNF1B*
rs8093601	18	51,772,473	C	G	1.064	imputed	X	*MBD2*
rs12956892	18	56,746,315	T	G	1.059	imputed	X	*LOC390858*
rs10460109	18	73,036,165	T	C	1.059	imputed	X	*TSHZ1*
rs8102476	19	38,735,613	C	T	1.069	genotyped	○	*DPF1/PPP1R14A*
rs2659124	19	51,354,597	T	A	1.156	imputed	○	*KLK3*

rs2735839	19	51,364,623	G	A	1.163	genotyped	○	*KLK3/KLK2*
rs6117562	20	753,310	A	G	1.093	imputed	○	*C20orf54*
rs12480328	20	49,527,922	T	C	1.221	imputed	○	*ADNP*
rs6062509	20	62,362,563	T	G	1.122	imputed	○	*ZGPAT*
rs138708	22	39,138,332	G	A	1.198	genotyped	○	*SUN2*
rs5759167	22	43,500,212	G	T	1.062	genotyped	○	*TTLL1/BIK*
rs2405942	X	9,814,135	A	G	1.080	imputed	○	*SHROOM2*
rs4826594	X	54,454,406	A	G	1.069	imputed	○	*TSR2*
rs4844289	X	70,407,983	G	A	1.051	imputed	○	*NLGN3/BCYRN1*

[a] Non-risk alleles were considered as reference.
