# Supplementary material for: 12 new susceptibility loci for prostate cancer identified by genome-wide association study in Japanese population
Source: Nat Commun. 2019 Sep 27;10:4422. doi: 10.1038/s41467-019-12267-6 (PMC6764957; doi:10.1038/s41467-019-12267-6)
Supplement: Supplementary file 3 — Description of Additional Supplementary Files [file 41467_2019_12267_MOESM3_ESM.pdf]

Description of Additional Supplementary Files

File Name: Supplementary Data 1

Description: Association of the reported SNPs in Japanese population
