# Supplementary material for: 12 new susceptibility loci for prostate cancer identified by genome-wide association study in Japanese population
Source: Nat Commun. 2019 Sep 27;10:4422. doi: 10.1038/s41467-019-12267-6 (PMC6764957; doi:10.1038/s41467-019-12267-6)
Supplement: Supplementary file 4 — Supplementary Data 1 [file 41467_2019_12267_MOESM4_ESM.pdf]

Supplementary Data 1   Association of the reported SNPs in Japanese population

SNP ID	Chr	Position	EffectAllele	NonEffectAllele	CASE_FREQ1	CTRL_FREQ1	RSQR	OR	*P* VALUE	Gene	Original SNP
rs636291	1	10556097	A	G	0.735	0.738	0.990	0.998	9.48.E-01	*PEX14*	
rs12139208[c]	1	88213014	T	C	0.756	0.767	1.000	0.971	2.95.E-01	LOC100505768	rs56391074
rs17599629	1	150658287	A	G	0.819	0.829	1.000	0.949	9.56.E-02	*GOLPH3L*	
rs1110165[c]	1	153913699	T	G	0.658	0.675	1.000	0.935	9.33.E-03	DENND4B	rs34579442
rs1218582	1	154834183	G	A	0.934	0.931	0.940	1.078	1.39.E-01	*KCNN3*	
rs4245739	1	204518842	A	C	0.964	0.959	1.000	1.154	2.73.E-02	*MDM4*	
rs1775148	1	205757824	C	T	0.526	0.514	0.928	1.046	7.72.E-02	*SLC41A1 3'*	
rs62106670	2	8597123	C	T	0.904	0.910	0.779	0.906	3.82.E-02	LINC00299	
rs11902236	2	10117868	C	T	0.886	0.892	1.000	0.951	1.97.E-01	*GRHL1*	
rs9287719	2	10710730	T	C	0.579	0.593	0.995	0.953	5.08.E-02	*NOL10*	
rs13385191	2	20888265	G	A	0.589	0.571	1.000	1.106	4.87.E-05	*C2orf43*	
rs1465618	2	43553949	T	C	0.676	0.647	0.959	1.137	1.15.E-06	*THADA*	
rs721048	2	63131731	G	A	0.953	0.955	1.000	0.936	2.58.E-01	*EHBP1*	
rs74702681	2	66652635	C	T			Monoallele			MEIS1-AS3	
rs10187424	2	85794297	T	C	0.650	0.634	1.000	1.083	1.60.E-03	*GGCX*	
rs11691517	2	111893096	T	G	0.810	0.803	0.7501	1.085	2.17.E-02	BCL2L11	
rs12621278	2	173311553	A	G	0.802	0.777	0.996	1.182	3.20.E-08	*ITGA6*	
rs34925593	2	174234547	T	C	0.562	0.579	0.9519	0.925	1.93.E-03	CDCA7	
rs2349070[c]	2	202130308	C	A	0.690	0.675	0.9903	1.056	4.02.E-02	CASP8	rs59308963
rs2292884	2	238443226	A	G	0.735	0.750	1.000	0.941	2.85.E-02	*MLPH*	
rs3771570	2	242382864	C	T	0.899	0.897	0.917	1.036	4.02.E-01	*FARP2*	
rs2660753	3	87110674	C	T	0.703	0.741	0.999	0.849	1.25.E-09	*VGLL3*	
rs2055109	3	87467332	T	C	0.875	0.893	0.885	0.837	8.97.E-06	*FOU1F1 5'*	
rs1283104	3	106962521	C	G	0.590	0.604	0.9998	0.959	8.63.E-02	LOC344595	
rs7611694	3	113275624	C	A	0.788	0.804	1.000	0.932	2.04.E-02	*SIDT1*	
rs10934853	3	128038373	C	A	0.492	0.484	1.000	1.006	8.10.E-01	*EEFSEC*	
rs6763931	3	141102833	G	A	0.631	0.642	0.999	0.951	4.61.E-02	*ZBTB38*	
rs182314334	3	152003952	T	C			Monoallele			MBNL1	
rs142436749	3	169092850	A	G			Monoallele			MECOM	
rs10936632	3	170130102	C	A	0.631	0.628	0.945	0.954	6.79.E-02	*SKIL/CLDN11*	
rs78943174	3	175252736	C	T			Mono allele			NAALADL2	
rs10009409	4	73855253	T	C	0.564	0.556	0.973	1.034	1.85.E-01	*COX18*	
rs1894292	4	74349158	G	A	0.680	0.676	0.858	1.031	2.75.E-01	*AFM*	
rs12500426	4	95514609	C	A	0.546	0.562	0.992	0.951	4.35.E-02	*PDLIM5*	
rs17021918	4	95562877	C	T	0.631	0.622	0.999	1.014	5.91.E-01	*PDLIM5*	
rs7679673	4	106061534	A	C	0.794	0.805	0.987	0.955	1.30.E-01	*TET2*	
rs2242652	5	1280028	G	A	0.787	0.756	0.940	1.146	7.78.E-06	*TERT*	
rs12653946	5	1895829	C	T	0.503	0.555	0.999	0.816	1.16.E-16	*IRX4*	
rs2121875	5	44365545	C	A	0.504	0.495	1.000	1.020	4.22.E-01	*FGF10*	

rs35148638	5	86610989	A	C	0.679	0.676	1.000	1.040	1.38.E-01	RASA1	
rs10793821	5	133836209	T	C	0.722	0.712	0.707	1.054	1.02.E-01	PHF15	
rs76551843	5	169171883	A	G			Monoallele			DOCK2	
rs6869841	5	172939426	C	T	0.829	0.830	0.999	1.021	5.20.E-01	*STC2/BOD1*	
rs4976790	5	177968915	G	T	0.939	0.935	1.000	1.073	1.62.E-01	COL23A1	
rs4713266	6	11219030	T	C	0.762	0.771	0.977	0.949	7.49.E-02	*NEDD9*	
rs115457135	6	30073776	G	A	0.691	0.691	0.000	0.000	6.70.E-01	*TRIM31*	
rs12665339	6	30600982	A	G			No data			ATAT1	
rs1966[c]	6	31107733	C	T	0.656	0.640	1.000	1.010	7.12.E-01	*CCHCR1*	rs130067
rs114376585[c]	6	32192331	G	*A*	0.858	0.855	0.092	1.129	2.88.E-01	*NOTCH4*	rs3096702
rs115306967	6	32400939	G	C	0.789	0.789	0.000	0.004	6.69.E-02	*HLA-DRB6*	
rs9296068	6	32988445	T	G			No data				
rs9469899	6	34793124	G	A	0.667	0.666	0.987	0.973	2.98.E-01	UHRF1BP1	
rs1983891	6	41536427	C	T	0.555	0.581	0.998	0.873	4.53.E-08	*FOXP4*	
rs4711748	6	43694598	T	C	0.541	0.532	0.958	1.063	1.42.E-02	LOC100132242	
rs9443189	6	76495882	A	G	0.698	0.679	0.998	1.097	4.68.E-04	*MYO6*	
rs2273669	6	109285189	A	G	0.919	0.924	0.944	0.926	1.01.E-01	*ARMC2*	
rs339331	6	117210052	T	C	0.680	0.631	1.000	1.222	8.66.E-15	*RFX6*	
rs1933488	6	153441079	G	A	0.796	0.804	1.000	0.937	3.19.E-02	*RGS17*	
rs4646284	6	160581543	-	G			No data			SLC22A1/SLC22A3	
rs9364554	6	160833664	C	T	0.659	0.672	1.000	0.950	4.49.E-02	*SLC22A3*	
rs138004030	6	170475879	G	A	0.848	0.849	0.072	0.934	5.96.E-01	LOC100505903	
rs527510716	7	1944287	G	C			No data			MAD1L1	
rs11452686	7	20413861	-	A			No data			ITGB8	
rs12155172	7	20994491	G	A	0.789	0.806	0.997	0.922	7.10.E-03	*LINC01162/SP8*	
rs10486567	7	27976563	A	G	0.882	0.901	0.989	0.837	5.58.E-06	*JAZF1*	
rs17621345	7	40875192	A	C	0.979	0.971	1.000	1.245	6.98.E-03	C7orf10	
rs56232506	7	47437244	G	A	0.696	0.698	0.927	1.029	2.95.E-01	*TNS3*	
rs6465657	7	97816327	C	T	0.899	0.895	0.999	1.054	1.83.E-01	*LMTK2*	
rs2928679	8	23438975	G	A	0.898	0.908	1.000	0.890	4.04.E-03	*NKX3.1*	
rs1512268	8	23526463	C	T	0.575	0.630	1.000	0.776	3.26.E-24	*NKX3.1*	
rs11135910	8	25892142	C	T	0.979	0.975	1.000	1.143	1.12.E-01	*EBF2*	
rs12543663	8	127924659	A	C	0.918	0.923	0.983	0.965	4.27.E-01	*PCAT1*	
rs10086908	8	128011937	T	C	0.833	0.797	1.000	1.269	7.47.E-14	*8q24(Block1)*	
rs16901979	8	128124916	C	A	0.748	0.812	0.999	0.700	3.44.E-34	*PRNCR1/8q24(Region2)*	
rs620861	8	128335673	G	A	0.555	0.537	0.945	1.061	1.82.E-02	*8q24(Block3/Region3)*	
rs6983267	8	128413305	T	G	0.627	0.666	1.000	0.865	1.22.E-08	*8q24(Block4/Region3)*	
rs1447295	8	128485038	C	A	0.758	0.821	0.993	0.664	4.10.E-42	*CASC6*	
rs7837688	8	128539360	G	T	0.775	0.842	0.832	0.579	2.02.E-58	*8q24(Block5/Region1)*	
rs1048169	9	19055965	T	C	0.862	0.867	0.937	0.981	6.07.E-01	HAUS6	
rs17694493	9	22041998	C	G	0.986	0.984	0.994	1.114	2.89.E-01	*CDKN2B-AS1*	
rs10122495	9	34049779	A	T	0.607	0.601	0.607	0.983	6.03.E-01	UBAP2	

rs817826	9	110156300	T	C	0.941	0.947	0.999	0.922	1.26.E-01	*RAD23B/KLF4*	
rs1571801	9	124427373	G	T	0.958	0.956	0.991	1.058	3.53.E-01	*DAB2IP*	
rs1182	9	132576060	C	A	0.714	0.723	0.999	0.941	2.53.E-02	TOR1A	
rs141536087	10	854442	-	CGCA			No data			LARP4B	
rs76934034	10	46082985	T	C			Mono allele			MARCH8	
rs10993994	10	51549496	C	T	0.510	0.545	0.998	0.859	4.60.E-10	*MSMB*	
rs1935581	10	90195149	C	T	0.481	0.461	0.999	1.060	1.66.E-02	RNLS	
rs3850699	10	104414221	A	G	0.905	0.899	1.000	1.034	4.14.E-01	*TRIM8*	
rs7094871	10	114712154	C	G	0.595	0.614	0.985	0.928	2.78.E-03	TCF7L2	
rs2252004	10	122844709	C	A	0.803	0.785	0.937	1.151	7.92.E-06	*FGFR2/WDR11*	
rs4962416	10	126696872	T	C			Mono allele			CTBP2	
rs1881502	11	1507512	C	T	0.932	0.936	0.968	0.977	6.46.E-01	MOB2	
rs7127900	11	2233574	G	A	0.904	0.909	0.999	0.949	2.10.E-01	*TH/ASCL2*	
rs61890184	11	7547587	G	A	0.853	0.863	0.968	0.871	9.64.E-05	PPFIBP2	
rs547171081	11	47421713	GG	-			No data			MIR4487	
rs1938781	11	58915110	A	G	0.675	0.696	0.999	0.896	3.00.E-05	*FAM111A*	
rs2277283	11	61908440	T	C	0.923	0.923	0.983	1.002	9.73.E-01	INCENP	
rs12785905	11	66951715	G	C			Monoallele			KDM2A	
rs7931342	11	68994497	T	G	0.767	0.775	1.000	0.939	3.03.E-02	*MYEOV/TPCN2*	
rs17245270[c]	11	76157489	A	C	0.857	0.846	0.999	1.115	1.62.E-03	C11orf30	rs11290954
rs11568818	11	102401661	T	C	0.934	0.933	1.000	1.042	3.96.E-01	*MMP7 5'*	
rs1800057	11	108143456	C	G			Monoallele			ATM	
rs11214775	11	113807181	G	A	0.707	0.697	1.000	1.022	4.18.E-01	*HTR3B*	
rs138466039	11	125054543	C	T			Monoallele			PKNOX2	
rs878987	11	134266372	A	G	0.962	0.965	0.678	0.884	1.14.E-01	B3GAT1	
rs2066827	12	12871099	T	G	0.974	0.974	0.859	1.041	6.25.E-01	CDKN1B	
rs10845938	12	14416918	G	A	0.692	0.695	0.776	1.010	7.31.E-01	GNAI2P1	
rs80130819	12	48419618	A	C			Mono allele			RP1-228P16.4/SENP1	
rs10875943	12	49676010	C	T	0.818	0.816	0.344	1.047	4.01.E-01	*TUB1C/LOC101927267*	
rs902774	12	53273904	T	C	0.046	0.059	1.000	1.142	1.96.E-02	*KRT78/KRT8*	
rs7968403	12	65012824	T	C	0.839	0.830	0.994	1.103	3.31.E-03	RASSF3	
rs5799921	12	90160281	-	A			No data			LOC107984543	
rs1270884	12	114685571	G	A	0.793	0.808	0.938	0.915	4.56.E-03	*TBX5/RBM19*	
rs7295014	12	133067989	G	A	0.880	0.872	0.958	1.125	1.93.E-03	FBRSL1	
rs9600079	13	73728139	G	T	0.579	0.614	0.999	0.875	7.44.E-08	*KLF5 3'*	
rs1004030	14	23305649	T	C	0.656	0.653	0.988	1.007	7.75.E-01	MMP14	
rs11629412	14	37138294	C	G	0.727	0.711	0.993	1.088	1.85.E-03	PAX9	
rs8008270	14	53372330	T	C			Mono allele			FERMT2	
rs7153648	14	61122526	G	C	0.707	0.713	0.958	0.933	1.07.E-02	*SIX1 10 kb 5’*	
rs58262369	14	64693912	C	T	0.921	0.934	0.993	0.831	7.16.E-05	*ESR2*	
rs7141529	14	69126744	C	T	0.858	0.858	1.000	1.010	7.80.E-01	*RAD51B*	
rs8014671	14	71092256	A	G	0.631	0.650	1.000	0.922	1.34.E-03	*TTC9 16 kb 5’*	

rs4924487	15	40922915	G	C	0.688	0.702	0.999	0.946	3.59.E-02	CASC5	
rs33984059	15	56385618	A	G			Monoallele			RFX7	
rs112293876	15	66764392	A	-			No data			MAP2K1	
rs11863709	16	57654326	C	T			Monoallele			ADGRG1	
rs12051443	16	71691329	A	G	0.673	0.670	0.980	1.026	3.35.E-01	*PHLPP2*	
rs201158093	16	82178644	A	-			No data				
rs684232	17	618965	C	T	0.508	0.489	1.000	1.077	2.20.E-03	*VPS53*	
rs28441558	17	7802868	T	C			No data			CHD3	
rs142444269	17	30098749	T	C	0.704	0.714	0.923	0.955	9.69.E-02	LOC100420482	
rs11649743	17	36074979	G	A	0.732	0.703	0.992	1.147	5.82.E-07	*HNF1B*	
rs4430796	17	36098040	A	G	0.706	0.648	0.859	1.365	5.49.E-27	*HNF1B*	
rs2680708	17	56456120	G	A	0.603	0.602	0.965	1.015	5.47.E-01	RNF43	
rs1859962	17	69108753	T	G	0.973	0.974	0.022	1.219	7.04.E-01	*CASC17*	
rs8093601	18	51772473	C	G	0.792	0.780	0.999	1.064	3.86.E-02	MBD2	
rs28607662	18	53230609	T	C			Monoallele			TCF4	
rs12956892	18	56746315	G	T	0.472	0.483	0.990	0.945	2.21.E-02	LOC390858	
rs533722308	18	60960944	-	T			No data			BCL2	
rs10460109	18	73036165	C	T	0.643	0.654	0.997	0.944	2.52.E-02	TSHZ1	
rs7241993	18	76773973	C	T	0.635	0.627	0.999	1.027	2.89.E-01	*SALL3/ATP9B*	
rs11666569	19	17214073	C	T	0.679	0.665	0.999	1.052	5.35.E-02	MYO9B	
rs118005503	19	32167803	G	C	0.971	0.964	0.811	1.163	5.70.E-02	LOC100507550	
rs8102476	19	38735613	T	C	0.606	0.627	0.999	0.935	7.28.E-03	*DPF1/PPP1R14A*	
rs11672691	19	41985587	G	A	0.509	0.509	0.920	1.020	4.33.E-01	*PCAT19*	
rs61088131	19	42700947	T	C	0.841	0.839	0.762	1.026	5.03.E-01	DEDD2	
rs2659124	19	51354597	T	A	0.641	0.605	0.972	1.156	1.69.E-08	KLK3	
rs2735839	19	51364623	G	A	0.639	0.600	0.992	1.163	2.85.E-09	*KLK3/KLK2 (PSA)*	
rs103294	19	54797848	T	C	0.752	0.746	0.998	1.005	8.67.E-01	*LILRA3*	
rs11480453	20	31347263	-	A			No data				
rs12480328	20	49527922	T	C	0.947	0.939	0.989	1.221	1.88.E-04	*ADNP*	
rs6068688[c]	20	52456926	A	T	0.598	0.606	0.999	0.967	1.81.E-01	SUMO1P1	rs6091758
rs2427345	20	61015611	C	T	0.793	0.790	1.000	1.007	8.20.E-01	*GATA5/RBBP8NL*	
rs6062509	20	62362563	G	T	0.594	0.611	0.998	0.891	3.86.E-06	*ZGPAT*	
rs1041449	21	42901421	A	G	0.884	0.885	0.923	1.003	9.39.E-01	*TMPRSS2*	
rs2238776	22	19757892	G	A	0.586	0.583	0.733	1.051	8.94.E-02	*TBX1*	
rs9625483	22	28888689	G	A			Monoallele			TTC28	
rs58133635	22	40471188	C	T	0.949	0.953	0.655	0.903	1.39.E-01	*TNRC6B*	
rs5759167	22	43500212	G	T	0.678	0.660	0.999	1.062	1.90.E-02	*TTLL1/BIK*	
rs2405942	X	9814135	A	G	0.891	0.870	0.998	1.080	4.99.E-03	*SHROOM2*	
rs17321482	X	11482384	C	T			Monoallele			ARHGAP6	
rs5945619	X	51241672	T	C	0.904	0.913	0.995	0.955	1.20.E-01	*NUDT10/NUDT11*	
rs2807031	X	52896949	T	C	0.970	0.970	0.818	1.013	8.23.E-01	*XAGE3*	
rs5919432	X	67021550	C	T			Mono allele			AR 3'	

rs6625711	X	70139850	A	T	0.464	0.451	0.877	1.025	1.90.E-01	*SLC7A/TEX11*
rs4844289	X	70407983	G	A	0.750	0.735	0.981	1.051	1.23.E-02	*NLGN3/BCYRN1*

[a]RSQR, imputation accuracy. SNPs were imputed in the GWAS.

[b]Non effect alleles were considered as reference.

[c]Original SNP was not contained the GWAS data. Each SNP indicated strong association ($r^2 \geq 0.8$) with original SNPs in the Japanese population in 1,000 genomes data.
