# Supplementary material for: 12 new susceptibility loci for prostate cancer identified by genome-wide association study in Japanese population
Source: Nat Commun. 2019 Sep 27;10:4422. doi: 10.1038/s41467-019-12267-6 (PMC6764957; doi:10.1038/s41467-019-12267-6)
Supplement: Supplementary file 5 — Reporting Summary [file 41467_2019_12267_MOESM5_ESM.pdf]

nature research

Corresponding author(s): Ryo Takata, Shusuke Akamatsu, and Hidewaki Nakagawa

Last updated by author(s): Aug 26, 2019

Reporting Summary

Nature Research wishes to improve the reproducibility of the work that we publish. This form provides structure for consistency and transparency in reporting. For further information on Nature Research policies, see Authors & Referees and the Editorial Policy Checklist.

Statistics

For all statistical analyses, confirm that the following items are present in the figure legend, table legend, main text, or Methods section.

n/a	Confirmed	
☐	☒	The exact sample size (*n*) for each experimental group/condition, given as a discrete number and unit of measurement
☐	☒	A statement on whether measurements were taken from distinct samples or whether the same sample was measured repeatedly
☐	☒	The statistical test(s) used AND whether they are one- or two-sided<br>*Only common tests should be described solely by name; describe more complex techniques in the Methods section.*
☐	☒	A description of all covariates tested
☐	☒	A description of any assumptions or corrections, such as tests of normality and adjustment for multiple comparisons
☐	☒	A full description of the statistical parameters including central tendency (e.g. means) or other basic estimates (e.g. regression coefficient) AND variation (e.g. standard deviation) or associated estimates of uncertainty (e.g. confidence intervals)
☐	☒	For null hypothesis testing, the test statistic (e.g. *F*, *t*, *r*) with confidence intervals, effect sizes, degrees of freedom and *P* value noted<br>*Give P values as exact values whenever suitable.*
☒	☐	For Bayesian analysis, information on the choice of priors and Markov chain Monte Carlo settings
☒	☐	For hierarchical and complex designs, identification of the appropriate level for tests and full reporting of outcomes
☐	☒	Estimates of effect sizes (e.g. Cohen's *d*, Pearson's *r*), indicating how they were calculated

Our web collection on statistics for biologists contains articles on many of the points above.

Software and code

Policy information about availability of computer code

Data collection	We used publicly available software for data collection. The software used is shown in the method section of our manuscript.
Data analysis	All statistical packages used are publicly available and referenced in the manuscript. We use MaCH, minimac, UnifiedGenotyper and HaplotypeCaller of GATK, VCMM (ver. 1.0.2), P-link and R for data analysis.

For manuscripts utilizing custom algorithms or software that are central to the research but not yet described in published literature, software must be made available to editors/reviewers. We strongly encourage code deposition in a community repository (e.g. GitHub). See the Nature Research guidelines for submitting code & software for further information.

Data

Policy information about availability of data

All manuscripts must include a data availability statement. This statement should provide the following information, where applicable:

- Accession codes, unique identifiers, or web links for publicly available datasets
- A list of figures that have associated raw data
- A description of any restrictions on data availability

GWAS summary statistics of prostate cancer will be publicly available at our website (JENGER, http://jenger.riken.jp/en/) and the National Bioscience Database Center (NBDC, https://humandbs.biosciencedbc.jp/en/) Human Database. Genotype data of case samples are available at NBDC under research ID hum0014.

Field-specific reporting

Please select the one below that is the best fit for your research. If you are not sure, read the appropriate sections before making your selection.

☒ Life sciences ☐ Behavioural & social sciences ☐ Ecological, evolutionary & environmental sciences

For a reference copy of the document with all sections, see nature.com/documents/nr-reporting-summary-flat.pdf

Life sciences study design

All studies must disclose on these points even when the disclosure is negative.

Sample size	We aimed to create the largest possible sample size in the Japanese population for the meta-analysis to gain the statistical power.
Data exclusions	We excluded the samples and variants based on the standard quality control procedure in GWAS. Detailed information on quality controls were sufficiently described in the method section of our manuscript.
Replication	Association study was conducted in two different sets of Japanese subjects and the results were combined using meta-analysis. We checked the directional consistency for the significant loci.
Randomization	Since we conducted case-control genetic study, we did not randomly allocated subjects into groups.
Blinding	Since we conducted case-control genetic study, blinding is not relevant to our study.

Reporting for specific materials, systems and methods

We require information from authors about some types of materials, experimental systems and methods used in many studies. Here, indicate whether each material, system or method listed is relevant to your study. If you are not sure if a list item applies to your research, read the appropriate section before selecting a response.

Materials & experimental systems

n/a	Involved in the study	
☒	☐	Antibodies
☒	☐	Eukaryotic cell lines
☒	☐	Palaeontology
☒	☐	Animals and other organisms
☐	☒	Human research participants
☒	☐	Clinical data

Methods

n/a	Involved in the study	
☒	☐	ChIP-seq
☒	☐	Flow cytometry
☒	☐	MRI-based neuroimaging

Human research participants

Policy information about studies involving human research participants

Population characteristics	GWAS included 5,088 cases from BioBank Japan, which was established in the Institute of Medical Science at the University of Tokyo16,17). Among the 5,088 cases for the GWAS, 272 (5.3%) subjects had a family history of PCa, 1,219 (23.9%) subjects revealed PSA ≥10 and 1,989 (39.1%) subjects were diagnosed to have cancer with Gleason score of 7 or higher. From the BBJ, pathologically proven PCa cases were selected. Non-cancer controls were from three population-based cohorts, including the J-MICC study, the JPHCStudy, IMM and ToMMo
Recruitment	Project participants were recruited from 66 hospitals, which consisted of 12 cooperating medical institutions located throughout Japan.
Ethics oversight	The ethical committees at all collaborating institutions and RIKEN approved the study.

Note that full information on the approval of the study protocol must also be provided in the manuscript.
